# Chromosome r(10)(p15.3q26.12) in a newborn child: case report

**DOI:** 10.1186/1755-8166-2-25

**Published:** 2009-12-07

**Authors:** Cecilia Gunnarsson, Barbara Graffmann, Jon Jonasson

**Affiliations:** 1 Division of Pathology and Clinical Genetics, Department of Clinical and Experimental Medicine, Linköping University, University Hospital, S-581 85 Linköping, Sweden; 2 Division of Pediatrics, Department of Clinical and Experimental Medicine, Linköping University, University Hospital, S-581 85 Linköping, Sweden

## Abstract

**Background:**

Ring chromosome 10 is a rare cytogenetic finding. Of the less than 10 reported cases we have found in the literature, none was characterized using high-resolution microarray analysis. Ring chromosomes are frequently unstable due to sister chromatid exchanges and mitotic failures. When mosaicism is present, the interpretation of genotype-phenotype correlations becomes extremely difficult.

**Results:**

We report on a newborn girl with growth retardation, microcephaly, congenital heart defects, dysmorphic features and psychomotor retardation. Karyotyping revealed a non-mosaic apparently stable ring chromosome 10 replacing one of the normal homologues in all analyzed metaphases. High-resolution oligonucleotide microarray analysis showed a *de novo *approximately 12.5 Mb terminal deletion 10q26.12 -> qter and a corresponding 285 kb terminal deletion of 10pter -> p15.3.

**Conclusion:**

This case demonstrates that an increased nuchal translucency thickness detected by early ultrasonography should preferably lead to not only QF-PCR for the diagnosis of Down syndrome but also karyotyping. In the future, microarray analysis, which needs further evaluation, might become the method of choice. The clinical phenotype of our patient was in agreement with that of patients with a terminal 10q deletion. For the purpose of genotype-phenotype analysis, there seems to be no need for a "ring syndrome" concept.

## Background

Ring chromosome 10 is a rare cytogenetic finding. Only a few cases have been reported and molecular cytogenetic definition of the aberrations is generally lacking. Common clinical features in these patients include growth retardation, microcephaly, facial dysmorphism, congenital malformations, and learning disability [1-6]. Presumably, small ring chromosomes representing loss of the major part of chromosome 10 only exist as supernumerary chromosomes. The partial trisomy is usually associated with growth retardation and microcephaly. Nearly full-length ring chromosomes 10 show deletions of the terminal segments of the short and long arms but could presumably also contain duplicated segments [7]. It seems likely that patients with nearly full length ring chromosomes 10 having lost e.g. the distal part of the long arm of the chromosome will have a phenotype similar to that seen in patients with terminal deletion of chromosome 10q as reviewed in Courtens et al. [8]. It remains to be found out whether ring chromosome phenotypes are dependent on the position and size of the deleted/duplicated segments only or represent a more general "ring syndrome" as discussed by Kosztolányi [9] and characterized by growth retardation, mental retardation and mild dysmorphic features. In this context, a confounding factor is that ring chromosomes are usually unstable due to sister chromatid exchanges and chromatid segregation errors during mitosis. Cells showing loss of the ring, the presence of double rings, isochromosomes, or other marker chromosomes derived from the ring are often present [10]. Such mosaicism may make the interpretation of genotype-phenotype correlations extremely difficult.

We here describe the clinical features associated with an apparently stable ring chromosome 10 replacing one of the normal homologues in a newborn girl. High-resolution oligonucleotide microarray hybridization was used for detailed evaluation of genomic gains and losses.

## Case presentation

The proband was born at 38+5 weeks of gestation as the second child of a 27-year-old mother and the first child of a 26-year-old father. The parents were unrelated and had no family history of chromosomal aberration or malformation. The pregnancy and labour had been uneventful apart from an increased nuchal translucency thickness of 4.1 mm, but no other malformations, detected at 13 weeks gestation by detailed ultrasound examination. Amniocentesis was performed and the amniotic fluid examined by QF-PCR (13,18,21, X and Y) with a normal female result.

**Figure 1:**
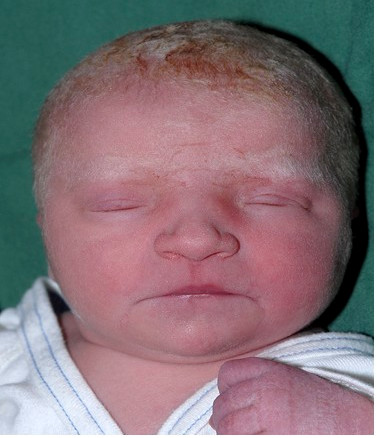
**Facial features of the proband in the newborn period**.

**Figure 2:**
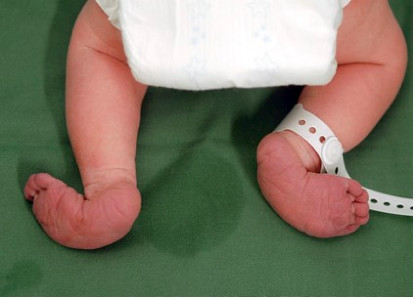
**Malformed "rocker bottom" feet**.

The newborn girl had growth retardation; birth weight 2570 g (-2 SD), length 43 cm (-3 SD), and microcephaly with head circumference 31 cm (-2 SD). Several dysmorphic features were noted (Fig. 1) including hypertelorism, small eyes, low set ears, prominent nasal bridge and stubby nose, small mouth with thin lips, short and webbed neck, widely spaced nipples, bilateral clinodactyly of the fifth finger, and malformed "rocker-bottom" feet (Fig. 2).

X-ray examination showed an enlarged heart but normal lung parenchyma. Ultrasound examination of the heart showed pulmonary hypertension, a large patent ductus arteriosus with bidirectional flow, a relatively large atrial septal defect (ASD) secundum and a small ventricular septal defect (VSD). The girl was transferred to a children's heart specialist centre where she when she was 2 months old was operated upon with closure of the VSD with a Gortex patch, direct suture of the ASD and closure of ductus Botalli.

Ultrasound examination of the kidneys revealed that they were small for age (at the lower reference interval value) but otherwise of normal appearance. Kidney growth was retarded and their size at 10 months is -2 SD. The other visceral organs have shown normal size, structure and ecogenicity upon ultrasound examination.

MRT brain at the age of 2 months was normal.

Eye examination did not show any sign of retinal disease or hypoplasia of nervus opticus. She had a slight ptosis and a smaller pupil on the right side, indicating Horner syndrome.

At 10 months she is a quiet hypotonic child who shows few spontaneous movements. She mostly lies on her back, but she is able to turn to one side with no supporting function from her arms. She can hold her head a few seconds. A nasogastric feeding tube has had to be introduced due to nutrition problems. She has a few sounds but no words yet and seldom gives a smile. She lives at home with her parents. She receives regular physiotherapy and has contact with a speech therapist as well as with a socio-therapist under the auspices of the children's habilitation unit.

## Results

### Cytogenetics

**Figure 3:**
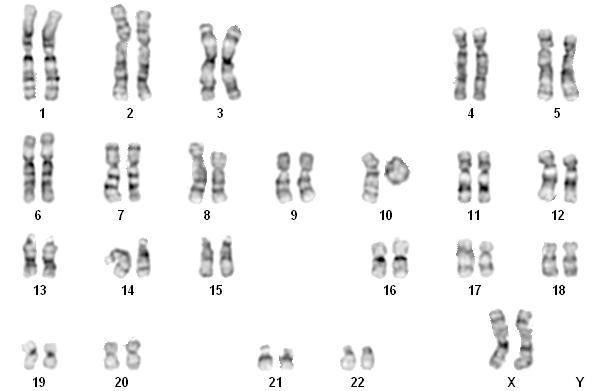
**Karyotype with ring chromosome 10 replacing one normal homologue**.

Cytogenetic analysis revealed a 46,XX, r(10) karyotype (Fig. 3). The ring chromosome 10 was present in all the 15 cells analysed. Due to the low number of metaphases the analysis could not be extended to a larger number of cells. Break points were difficult to assign but the results indicated a deletion of the distal part of the long arm of chromosome 10 in the ring formation. Both parents had a normal karyotype 46,XX and 46,XY, respectively.

### Microarray hybridization

**Figure 4:**
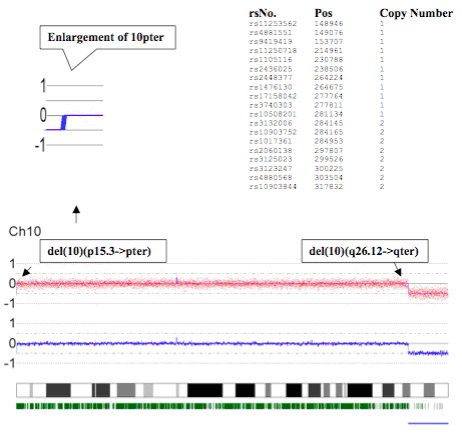
**Microarray hybridization for high-resolution mapping of break points on ring chromosome 10**. Copy number loss "partial monosomy" was recorded for the short arm of Chr 10: (0-) 148,946 - 281,134, corresponding to cytobands 10pter-> p15.3. An enlarged insert with the corresponding CNAG copy number analysis results presented as a table is shown above. A larger terminal deleted region is seen on the right hand side (i.e. loss of 1 copy) of the long arm of Chr 10: 122,736,794 - 135,259,604, which corresponds to cytoband 10q26.12->qter. The SNP analysis shown below the chromosome 10 ideogram indicates loss of heterozygosity in this region (straight blue bar at bottom right), confirming the copy number results.

Microarray hybridization analysis was used for high-resolution mapping of break points (Fig. 4). A copy number loss ("partial monosomy") was recorded for the short arm of chromosome 10: position (0-) 148,946 - 281,134, corresponding to cytobands 10pter -> p15.3, as well as for the long arm of chromosome 10: position 122,736,794 - 135,259,604, corresponding to cytobands 10q26.12 -> qter. An apparently unrelated gain in copy number of chromosome 10: position 46,363,383-47,154,881 (Fig. 4) was considered to be a copy number variation (CNV), without clinical significance.

## Discussion

This case demonstrates clearly the importance of performing not only QF-PCR for the diagnosis of Down syndrome but also a full cytogenetic investigation when a substantially increased nuchal translucency thickness is detected by early ultrasonography. The chromosomal aberration would then have been revealed by prenatal cytogenetic diagnosis. However, the appropriate evaluation of ring chromosomes requires molecular karyotyping because assignment of breakpoints on ring chromosomes is often difficult and at best only approximate on GTG-banded cytogenetic preparations. Molecular techniques will also complement cytogenetic investigation of the mosaicism that is often present due to loss or rearrangement of the ring chromosome. In the present case, using oligonucleotide microarray hybridization we were able to exclude any major degree of mosaicism and also to identify both breakpoints.

### Chromosome 10q26 deletion syndrome?

It seems that the chromosome 10q26 deletion syndrome OMIM #609625 [11] provides a good description of the clinical features of our patient. Irving et al. [12] reported 8 familial and 4 de novo cases of terminal 10q deletion syndrome, and 3 cases with interstitial deletion of 10q25.2-q26.3. Common features included facial asymmetry, prominent nose, thin upper lip, strabismus, low birth weight, short stature and fifth finger clinodactyly. Variable degrees of learning difficulty were found in 11 patients and 4 had seizures. Four patients had violent mood swings and aggression, whereas 2 had affectionate behaviour. Visceral abnormalities were also noted: 3 patients had renal anomalies, 2 with vesico-ureteral reflux and 1 with acute renal failure of unknown etiology and 2 patients had an ASD. Courtens et al. [8] described a girl with a 6.1 Mb subterminal 19q26.2 deletion and a similar phenotype to the present case. They made an extensive literature review of "pure" subterminal 10q25-q26 and terminal 10q26 deletion. Psychomotor retardation, postnatal growth retardation, strabismus, prominent/broad nasal bridge, low-set malformed large posteriorly rotated ears, hypertelorism, microcephaly at birth, congenital heart disease, small for gestational age at birth were the most common features. Thus, to "explain" the phenotype in the present case there seems to be no need for a more general "ring syndrome" concept proposed by Kosztolányi [9] and characterized by growth retardation, mental retardation and mild dysmorphic features.

### Genes of importance lost in the ring chromosome formation

The 12.5 Mb long deletion of the long arm from cytoband 10q26.12 -> qter represents in the order of 100 protein coding genes. Therefore, one could hardly judge the contribution of the missing copy of the individual gene to the observed phenotype. However, one gene, *FGFR2 *seems particularly important to discuss in connexion with birth defects. The protein structure coded by this gene constitutes three immunoglobulin-like domains in an extra-cellular region, a single membrane-spanning segment, and a cytoplasmic tyrosine kinase domain. Mutations cause several dominantly inherited congenital skeletal disorders: Crouzon syndrome, Pfeiffer syndrome, isolated coronal synostosis, Apert syndrome, Jackson-Weiss syndrome, Beare-Stevenson cutis gyrata syndrome. These syndromes are also associated with other phenotypes such as limb, cardiac, CNS and tracheal malformations. Apert, Pfeiffer, Crouzon, and Jackson-Weiss syndromes are due to gain-of-function mutations of *FGFR2 *in either the Ig II-III linker region (Apert) or the Ig III domain. Our patient did not have features suggesting any one of these syndromes, which could be due to the fact that loss of one *FGFR2 *allele will presumably result in haplo-insufficiency rather than gain-of-function. As regards the other genes having lost one allele in the ring formation, one could speculate that the *ATE1 *gene might be a likely candidate to explain the cardiac anomalies. This gene encodes an arginyltransferase, an enzyme that is involved in posttranslational conjugation of arginine to N-terminalaspartate or glutamate residues. In an animal model about 70% of knockout Ate1 knockout mice had persistent truncus arteriosus, with the common root of the aorta and pulmonary artery straddling a large ventral septal defect [13].

Only two protein-coding genes, *TUBB8 *and *ZMYND11 *have been localized to the lost segment of the short arm of chromosome 10. The functional *TUBB8 *gene is thought to have arisen by duplication of a subtelomeric beta-tubulin gene on chromosome 4 [14]. The human genome contains several authentic beta-tubulin genes located subtelomeric on different chromosomes. Haplo-insufficiency is hardly to be expected for any one of these genes. Copy number variants have indeed been found [15] covering both the full *TUBB8 *coding genomic sequence and the *ZMYND11 *sequence that represents a hypothetical zinc finger protein; only identified at the transcript level. Thus, it is unlikely that the lost region 10pter -> p15.3 in the present ring chromosome would make a significant contribution to the girl's phenotype.

## Conclusion

Although the pattern of anomalies observed in the present case of ring chromosome 10 would not allow a specific chromosomal diagnosis on clinical grounds alone, the phenotype was in good agreement with the literature describing terminal 10q deletion and the suspicion of a chromosome anomaly was raised immediately after birth. The present data indicates a lack of a ring chromosome syndrome per se. Considering the consequences of performing QF-PCR instead of a full karyotype for prenatal diagnosis, the message is that substantially increased nuchal translucency should preferably imply genetic counselling and follow-up with karyotyping. It is worth mentioning the subsequent change of local recommendations for prenatal diagnosis in similar cases, which now include a full karyotype. In the future, microarray analysis, which needs further evaluation, might become the method of choice.

## Methods

### Cytogenetics

Conventional cytogenetic analysis on GTG-banded chromosomes from cultured lymphocytes was performed according to standard techniques. Karyotyping was performed with the aid of BandView^® ^software from Applied Spectral Imaging (Stockholm, Sweden).

### Microarray analysis

DNA was extracted from peripheral blood using BioRobot EZ1 (Qiagen, Solna, Sweden). High-resolution microarray analysis of 262,000 autosomal and X-chromosome SNPs was performed using GeneChip^® ^Human Mapping 250K Nsp SNP Array kit (Affymetrix, High Wycombe, UK) and CNAG version 2 analysis software. UCSC Genome Browser on Human May 2004 Assembly was used for localisation of gains and losses in the copy number analysis [16].

## Abbreviations

EEG: Electroencephalography; GTG-bands: G-bands after trypsin and giemsa staining; MRT: Magnetic resonance tomography; QF-PCR: Quantitative fluorescence polymerase chain reaction; SNP: Single nucleotide polymorphism.

## Consent

This case report is presented with the consent of the patient's parents.

## Competing interests

The authors declare that they have no competing interests.

## Authors' contributions

CG, BG and JJ wrote the manuscript; BG coordinated analysis of clinical features; CG signed out the molecular cytogenetic results; All authors have read and approved the manuscript.
